# Hypoglycemic effect of *Carica papaya* leaves in streptozotocin-induced diabetic rats

**DOI:** 10.1186/1472-6882-12-236

**Published:** 2012-11-28

**Authors:** Isela Esther Juárez-Rojop, Juan C Díaz-Zagoya, Jorge L Ble-Castillo, Pedro H Miranda-Osorio, Andrés E Castell-Rodríguez, Carlos A Tovilla-Zárate, Arturo Rodríguez-Hernández, Hidemi Aguilar-Mariscal, Teresa Ramón-Frías, Deysi Y Bermúdez-Ocaña

**Affiliations:** 1 Centro de Investigación, DACS, Universidad Juárez Autónoma de Tabasco (UJAT), Villahermosa, Tabasco, 86150, Mexico; 2 División de Investigación, Facultad de Medicina, UNAM, México, D F, Mexico; 3 Unidad de Medicina Familiar 10, Instituto Mexicano del Seguro Social, Xalapa, Veracruz, Mexico; 4 División Académica Multidisciplinaria de Comalcalco, Universidad Juárez Autónoma de Tabasco, Comalcalco, Tabasco, Mexico

**Keywords:** Diabetes, *Carica papaya*, Hypoglycemic action

## Abstract

**Background:**

Traditional plant treatment for diabetes has shown a surging interest in the last few decades. Therefore, the purpose of this study was to assess the hypoglycemic effect of the aqueous extract of *C. papaya* leaves in diabetic rats. Several studies have reported that some parts of the *C. papaya* plant exert hypoglycemic effects in both animals and humans.

**Methods:**

Diabetes was induced in rats by intraperitoneal administration of 60 mg/kg of streptozotocin (STZ). The aqueous extract of *C. papaya* was administered in three different doses (0.75, 1.5 and 3 g/100 mL) as drinking water to both diabetic and non-diabetic animals during 4 weeks.

**Results:**

The aqueous extract of *Carica papaya* (0.75 g and 1.5 g/100 mL) significantly decreased blood glucose levels (p<0.05) in diabetic rats. It also decreased cholesterol, triacylglycerol and amino-transferases blood levels. Low plasma insulin levels did not change after treatment in diabetic rats, but they significantly increased in non-diabetic animals. Pancreatic islet cells were normal in non-diabetic treated animals, whereas in diabetic treated rats, *C. papaya* could help islet regeneration manifested as preservation of cell size. In the liver of diabetic treated rats, *C. papaya* prevented hepatocyte disruption, as well as accumulation of glycogen and lipids. Finally, an antioxidant effect of *C. papaya* extract was also detected in diabetic rats.

**Conclusions:**

This study showed that the aqueous extract of *C. papaya* exerted a hypoglycemic and antioxidant effect; it also improved the lipid profile in diabetic rats. In addition, the leaf extract positively affected integrity and function of both liver and pancreas.

## Background

There are large randomized controlled trials that show the benefit of a tight blood glucose control which reduces microvascular and macrovascular complications, but despite this, many diabetics do not keep control of their blood glucose levels or this control is poorly done [1,2]. Poor and inadequate glycemic control constitutes a major public health problem and thus research on new substances with hypoglycemic properties is required. Medicinal plants are gradually gaining global acceptability given their potential as bioactive agents to be used as pharmaceuticals. New hypoglycemic agents derived from plants have shown both hypoglycemic action and the ability to improve some of the secondary complications of diabetes such as kidney damage, fatty liver, and oxidative stress. In addition, some tropical herbs offer both benefits as it has been recently informed in experimental models [3-5]. In this sense, several authors have reported an increase in the number of Mexican diabetic patients who employ medicinal plants to decrease blood glucose. Several studies have reported the existence of 306 plants or fruits used as herbal remedies for diabetes [3]. Among them lies *Carica papaya*, an herbaceous plant, member of the small family Caricaceae. This plant is widely cultivated for its edible pleasant fruit, which provides good nutritional value and easy digestion. Native of the lowlands of eastern Central America, it can be found from Mexico to Panama, though it is grown in all tropical and many subtropical regions of the world. Infusions made from different parts of the plant including leaves have been used as therapeutic remedies due to their medicinal properties [4]. There is evidence that *C. papaya* leaves reduce symptoms of asthma, worming and dysentery [5,6]. Moreover, papaya leaf extracts have long been used as remedy for cancer and infectious diseases [6]. The leaf aqueous extract accelerates wound healing [7,8], whereas the leaf methanol extract has exhibited vasodilating and antioxidant effects, both being associated with cardiovascular risk reduction [5].

Besides their hypoglycemic properties [3], different parts of *C. papaya* are used in Mexican folk medicine to treat various diseases such as diarrhea, inflammation and diabetes [8,9]. The present study was carried out to evaluate the hypoglycemic effect of the aqueous extract of *C. papaya* leaves in streptozotocin-induced diabetic rats.

## Methods

### Animals

Experiments were performed on adult male Wistar rats (body weight range 250–300 g), 10 to 11 weeks of age. Animals were housed and maintained at 22°C under a 12-h light/12-h dark cycle, with free access to food and water. Experiments were carried out during the normal light/dark cycle, always starting at the same hour (10:00 AM). Efforts were made to minimize animal suffering and to reduce the number of animals used. All experiments complied with the Guidelines on Ethical Standards for the investigation in animals; the study was approved by the local Internal Committee for the care and use of laboratory animals (001-10/CICUAL/DACS) División Académica de Ciencias de la Salud, (DACS), Universidad Juárez Autónoma de Tabasco, (UJAT).

### Chemical and plant products

Streptozotocin was purchased from Sigma (St Louis, MO, USA). All other chemicals of analytical grade were obtained from Merck. Kits for different enzyme assays were purchased from Biosystems S.A., Mexico. The leaves from *Carica papaya* were collected during June to September 2010 from Cintalapa, in the state of Chiapas, Mexico. The plant was authenticated at the Division Académica de Ciencias Biológicas (DACB) UJAT as *Carica papaya* and a voucher specimen is kept in its herbarium (No. 32307) at the DACB-UJAT in Tabasco, México.

### Induction of diabetes

Experimental diabetes [10] was induced following an overnight fast, by a single intraperitoneal injection of 60 mg/kg STZ freshly dissolved in distilled water. Control animals received 0.9% sterile saline. Hyperglycemia was confirmed 4 days after injection by measuring the tail vein blood glucose level with an Accu-Check Sensor Comfort glucometer (Roche, Mexico City). Only the animals with fasting blood glucose levels ≥ 250 mg/dL were selected for the study in accordance with the report by Gupta et al. [11]. This procedure induced hyperglycemia in 80% of the animals 4 days after they were injected; only 2 rats had to be discarded in this lot.

### *Carica papaya* leaf processing

*C. papaya* leaves were first washed in 1% iodine aqueous solution, followed by a distilled water wash and dried afterwards. Three leaf portions were used, weighing 3.5, 7.5 and 15 g. These were homogenized in water and filtered through a No.41 filter paper with 20–25 μm retention (Whatman) to mimic the traditional procedure used by people from southeast Mexico. Each extract containing 0.75, 1.5 or 3 g/100 mL. These doses were chosen in accordance with a preliminary pilot study that included from 15 to 60 g of leaves in 500 mL of water, mimicking the amounts used by *C. papaya* users. Animals of the diabetic and non-diabetic control groups received only drinking water. All animals were kept under the above mentioned experimental conditions for a 30-day period.

### Study design

In order to determine the hypoglycemic effect of *C. papaya* leaves in diabetic rats, oral doses of *C. papaya* aqueous extract (0.75, 1.5 y 3.0 g/100 mL) were administered as drinking water. Experimental non-diabetic rats also received similar doses of *C. papaya* leaf extract as drinking water. A total of 42 Wistar rats were used (24 diabetic and 18 non-diabetic). Diabetic rat groups included control, 0.75, 1.5 and 3.0 g/100 mL and non-diabetic rats included control, 0.75 and 1.5 g/100 mL. For each group of six rats (n=6), the necessary biochemical determinations were carried out to identify the pharmacological effects of *C. papaya* leaves*.* For all animal groups, water intake was determined daily and data are presented as the area under the curve in the water consumption graph along the 30 days of the experiment. Body weight was measured at baseline and every week. After the 4-week treatment and after a 12-h food withdrawal, rats were sacrificed by decapitation.

### Biochemical parameters

Blood was collected and serum was immediately frozen and stored at −70°C until the biochemical determinations were performed. Serum levels of glucose, cholesterol, triglycerides, high-density lipoprotein-cholesterol (HDL-C), aspartate aminotransferase (AST), alanine aminotransferase (ALT) and alkaline phosphatase (ALP), as well as total bilirubin and direct bilirubin were analyzed using a Clinical Chemistry System from Random Access Diagnostics. Plasma insulin concentrations were determined by an enzymatic immunoassay method (ELISA ALPACO insulin rat).

### NO (nitrate+nitrite) concentration

NO production was assessed by measuring the nitrate and nitrite concentrations according to the commercial kit from Cayman Chemicals, based on the nitrate conversion to nitrite using nitrate reductase followed by the Griess reaction. The final products of nitric oxide *in vivo* are nitrate and nitrite, hence the best index of total NO production is the sum of both compounds.

### Measurement of triglyceride and cholesterol levels in the liver

Liver lipids were extracted according to the method by Folch et al. [12], whereas triglyceride and cholesterol concentrations were measured using enzymatic colorimetric determinations according to diagnostic kits from Lakeside Laboratories.

### Pancreas and liver histopathological examination

The histopathologic analysis was performed by light microscopy. Liver and pancreas tissue sections were fixed in 10% buffered formalin. After fixation, the sample was washed with running water and processed to obtain 5 μm thick paraffin sections. Pancreas sections were stained with hematoxilin and eosin (HE), whereas liver sections were stained with both HE and Periodic Acid Schiff’s (PAS) methods. The pancreas histopathological analysis consisted in measuring the number and diameter of the islets of Langerhans.

### Statistics analysis

All data are expressed as mean ± S.E.M. One- or two-way analysis of variance (ANOVA) was performed followed by Tukey’s test to compare the differences between treatments. Differences were considered statistically significant for p< 0.05.

## Results

### Effect of the aqueous extract of *C. papaya* leaves on body weight and water ingestion of STZ-induced diabetic rats

Body weight was recorded every week and the final data are shown in Table 1. There was a significant decrease in body weight in diabetic rats (240±4.17 g) with respect to non-diabetic animals (290±2.88 g) (p< 0.001). The aqueous extract of *C. papaya* (0.75, 1.5 g/100 mL) caused a slight reduction in weight in diabetic rats (p<0.05) (Table 1). In contrast, non-diabetic rats showed an increase in body weight after treatment with respect to control non-diabetic rats (p< 0.05) (Table 1). Water consumption was higher in diabetic rats (1008±29.26 mL) than in non-diabetic animals (197±9.34 mL) (p< 0.001). In addition, the treatment of diabetic rats with 0.75 and 1.5 g/100 mL of *C. papaya* leaf extract significantly reduced water consumption (p < 0.05) (Table 1).

**Table 1:** **Effect of aqueous extract of *C. papaya *leaf on body weight and water consumption in diabetic and non-diabetic rats**

***C. papaya***		**Diabetic**			**Non-diabetic**		
**g/100 mL**	**Control**	**0.75**	**1.5**	**3**	**Control**	**0.75**	**1.5**
**Weight (g)**	240±4.17^a^	252±2.3	253.2±4.4	214.5±1.89^b^	290±2.88	373.1±28.55^c^	311.4±20.67^c^
**Water Consumption (mL)**	1008±29.26^a^	437.1±15.13^b^	333.9±19.61^b^	828.5±89.62^b^	197.0±9.34	165.1±7.88^c^	296.6±12.81^c^

Data are expressed as mean ± S.E.M; n=6. ANOVA followed by Tukey’s test (p< 0.05). ^a^statistically different from non-diabetic rats; ^b^statistically different from diabetic control and ^c^statistically different from non-diabetic control.

### Effect of the aqueous extract of *C. papaya* leaves on blood glucose levels

Streptozotocin-induced diabetes resulted in a significant increase in serum glucose levels (434±7.49 mg/dL) in comparison to the control group (99.5±6.4 mg/dL) (p< 0.001). After the administration of the different doses of the aqueous extract of *C. papaya* (0.75, 1.5 and 3 g/100 mL) to diabetic rats during 30 days a significant decrease in blood glucose levels (305.4±9.68; 306.±8.8 and 250± 10.2 vs. 434±7.49 mg/dL, respectively) (p< 0.001) was observed (Table 2).

**Table 2:** **Effect of aqueous extract of *C. papaya *leaf on biochemical parameters in diabetic and non-diabetic rats**

***C. papaya***	**Glucose**	**Chol**	**TAG**	**HDL-Chol**	**Insulin**	**NOx**
**(g/100 mL)**	**(mg/dL)**	**(mg/dL)**	**(mg/dL)**	**(mg/dL)**	**(ng/mL)**	**(NO**_**3**_**+NO**_**2**_**μM)**
**Diabetic**						
**Control**	434±7.49^a^	75.62 ±2.9^a^	232.1±17.3^a^	34.25±2.09	0.42±0.02^a^	18.29±3.75^a^
**0.75**	305.4±9.68^b^	70.32±3.27	146.5±8.9^b^	37.12±3.23	0.43±0.05^b^	26.24±3.47^b^
**1.5**	306.5±8.8^b^	88.11±4.56	144.5±11.6^b^	43.32±2.23	0.38±0.2^b^	35.68±3.74^b^
**3**	250±10.2^b^	57.37±2.9^b^	93.71±17.2^b^	27.2±1.09^b^	0.43±0.01^b^	27.96±3.2^b^
**Non-diabetic**						
**Control**	99.5±6.4	63.61±2.24	86.14±11.4	31.18±1.31	0.97±0.1	36.01±5.23
**0.75**	102.8±9.54	81.5±5.5^c^	208.2±12^c^	30.69±1.35	1.06±0.06	31.68±4.9
**1.5**	89.19±1.9	73.23± 3.3^c^	135.8±12.6^c^	30.14±1.69	1.29±0.17^c^	31.19±3.75

Data are expressed as mean ± S.E.M; n=6. ANOVA followed by Tukey’s test (p< 0.05). ^a^statistically different from non-diabetic rats; ^b^statistically different from diabetic control and ^c^statistically different from non-diabetic control.

### Effect of the aqueous extract of *C. papaya* leaves on serum cholesterol and triglycerides

Serum cholesterol levels in diabetic rats showed a significant rise in comparison to the values in non-diabetic control rats (75.62±2.9 vs. 63.61±2.24 mg/dL) (p< 0.05) (Table 2). Serum triacylglycerol levels were also elevated in diabetic rats compared to non-diabetic animals (232.1±17.3 and 86.14±11.4 mg/dL, respectively) (p< 0.001) (Table 2). On the other hand, non-diabetic animals receiving 0.75 and 1.5 g/100 mL of the *C. papaya* leaf extract showed a significant increase in serum cholesterol (81.5±5.5, 73.23±3.3 vs. 63.6±2.24 mg/dL) (p< 0.001) and triglycerides (208±12, 135±12.6 vs. 86.14±11.14) (p< 0.001) levels when compared to non-diabetic control rats (Table 2). After a 4-week administration of 3 g/100 mL of *C. papaya* leaf extract to diabetic rats a significant decrease in serum cholesterol levels was observed in comparison to untreated diabetic rats (57.37±2.9 vs. 75.62±2.9 mg/dL) (p< 0.001). Serum triacylglycerol levels were also decreased in a dose-dependent manner in *C. papaya* treated rats compared to diabetic controls (146.5±8.9, 144.5±11.6, 93.7±17.2 mg/dL vs. 232.1±17.3 mg/dL, respectively) (p< 0.001). High-density lipoprotein cholesterol levels were significantly increased with 1.5 g/100 mL of *C. papaya* leaf extract in diabetic rats in comparison to diabetic control (43.32±2.23 mg/dL vs. 34.25±2.09 mg/dL) (p< 0.05) (Table 2) and non-diabetic control rats.

### Determination of basal plasma insulin

Blood insulin levels were dramatically reduced in all groups of STZ-treated rats with or without *C. papaya* leaf extracts in the drinking water (0.42±0.2 ng/mL), when compared to non-diabetic control rats (0.97±0.1 ng/mL) (p< 0.001). Conversely, blood insulin levels were significantly increased in non-diabetic rats receiving 0.75 or 1.5 g/100 mL of *C. papaya* leaf extracts (1.06±0.06 and 1.29±0.17 ng/mL, respectively vs. 0.97±0.1 ng/mL) (p< 0.05) (Table 2).

### Nitric oxide (nitrate+nitrite) concentration

Nitric oxide (NO) production measured through the evaluation of its stable metabolites nitrate and nitrite was significantly low in diabetic rats (18.29±3.75 μM) when compared to non-diabetic animals (36.01±5.23 μM) (p< 0.001). On the other hand, the administration of 0.75 to 3 g/100 mL of *C. papaya* leaf extracts to diabetic rats significantly enhanced the amount of nitrite-nitrate in comparison to the untreated diabetic group (p<0.05) (Table 2).

### Biochemical parameters

Serum activities of ALT, AST and ALP, biomarkers of liver toxicity, were elevated in SZT-induced diabetic rats (276±16.6, 345±10.4, 798.4±14.7 U/L, respectively) when compared to non-diabetic controls (88.6±2.02, 285.3±7.9, 243.6±9.13 U/L, respectively) (p< 0.001) (Table 3). Treatment of diabetic rats with 0.75 and 1.5 g/100 mL of *C. papaya* leaf extract significantly reduced the activity of these biomarkers with respect to control diabetic rats (p< 0.001, p<0.001 and p< 0.001; respectively for both doses), but treatment with 3 g/100 mL did not reduce the activity of these serum enzymes in diabetic rats (Table 3). Similarly, the administration of 0.75 and 1.5 g/100 mL of *C. papaya* leaf extract significantly reduced the activities of AST and ALP (p <0.05) in non-diabetic rats compared to non-diabetic controls (Table 3).

**Table 3:** **Effect of aqueous extract of *C. papaya *leaf on the hepatic profile in diabetic and non-diabetic rats**

***C. papaya***	**ALT**	**AST**	**ALP**	**TB**	**DB**
**(g/100 mL)**	**(U/L)**	**(U/L)**	**(U/L)**	**(mg/dL)**	**(mg/dL)**
**Diabetic**					
**Control**	276±16.6^a^	345.5±10.4^a^	798.4±14.7^a^	0.69±0.11^a^	0.53±0.09^a^
**0.75**	61.16±2.9^b^	169.5±4.8^b^	441±10.6^b^	0.35±0.03^b^	0.31±0.02^b^
**1.5**	81.85±14.6^b^	187.9±14.1^b^	437.7±10.8^b^	0.63±0.07	0.52±0.08
**3**	314.9±13.7^b^	416.7±16^b^	642.6±11.6^b^	0.45±0.05^b^	0.44±0.08
**Non-diabetic**					
**Control**	88.6±2.02	285.3±7.9	243.6±9.13	0.35±0.03	0.25±0.02
**0.75**	66.4±6.3^c^	260.4±18.6^c^	136.1±3.7^c^	0.39±0.01	0.25±0.01
**1.5**	86.5±4.6	253.7±9.6^c^	109.6±5.5^c^	0.55±0.06^c^	0.38±0.04^c^

Data are expressed as mean ± S.E.M; n=6. ANOVA followed by Tukey’s test (p <0.05). ^a^statistically different from non-diabetic rats^; b^statistically different from diabetic control and ^c^statistically different from non-diabetic control.

### Effect of the aqueous extract of *C. papaya* leaves on hepatic cholesterol and triglycerides

Liver weight was lower in diabetic rats (8.37±0.69 g) than in non-diabetic animals (10.45±1.65 g) (p< 0.05). In the groups drinking the aqueous extract of *C. papaya* leaves, an increase in liver weight in diabetic rats was detected when compared to liver weight in untreated diabetic animals; however, statistical significance was only reached for the 0.75 g/100 mL dose (p<0.05). Liver triglycerides concentration was significantly increased in diabetic rats (69.71±5.00 mg/g tissue) in comparison to non-diabetic rats (60.39±3.5 mg/g tissue) (p< 0.05). *C. papaya* leaf-treated diabetic rats exhibited a decrease in liver triacylglyceride concentrations for the three extract doses (55.40±4.64, 59.07±6.48 and 56.47±2.43 mg/g tissue, respectively) (p<0.05), but no changes were observed in liver cholesterol concentrations in all groups (Table 4).

**Table 4:** **Effect of aqueous extract of *C. papaya *leaf on the hepatic lipids in diabetic and non-diabetic rats**

***C. papaya***	**LW**	**Chol**	**TAG**
**(g/100 mL)**	**(g)**	**(mg/g tissue)**	**(mg/g tissue)**
**Diabetic**			
**Control**	8.37±0.69^a^	10.78±1.06	69.71±5.00
**0.75**	9.88±0.89^b^	11.23±1.04^b^	55.40±4.64^b^
**1.5**	8.52±1.28	10.54±1.11^b^	59.07±6.48
**3**	9.15±1.12	9.49±0.82^b^	56.47±2.43^b^
**Non-diabetic**			
**Control**	10.45±1.65	9.66±1.11	60.39±3.5
**0.75**	10.88±1.8	10.25.1±7.88^c^	57.55±0.72
**1.5**	10.63±1.65	10.29±1.44^c^	56.4±3.86

Data are expressed as mean± S.E.M; n=6. ANOVA followed by Tukey’s test (p <0.05). ^a^statistically different from non-diabetic rats; ^b^statistically different from diabetic control and ^c^statistically different from non-diabetic control.

### Liver and pancreas histopathological examination

**Figure 1:**
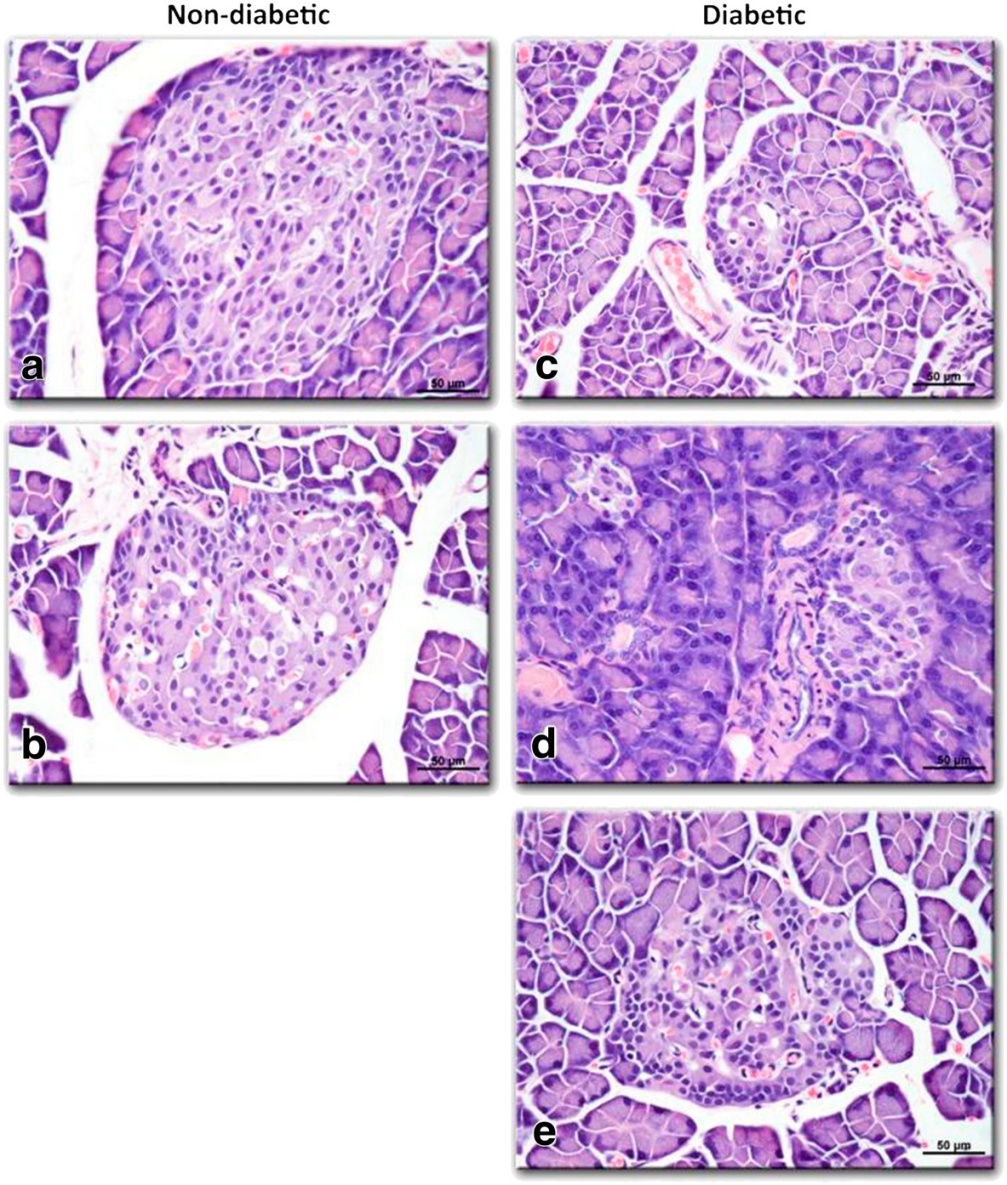
**Effect of aqueous extract of *C. papaya *leaves on the histological of rat pancreas evaluated by hematoxylin and eosin (H&E) staining (50X). a**) Shows the Normal aspect of a Langerhans islet. **b**) Pancreas of non-diabetic rat treated with 1.5 g/100 mL showing a reduced Langerhans islet with smaller size with respect to **a**). **c**) Diabetic rat pancreas without treatment showing a small Langerhans’ islet due to streptozotocin injury. In contrast, diabetic rats treated with *C. papaya* leaf extract showed larger Langerhans’ islets for all doses: (**d**) 0.75 g/100 mL **e**) 1.5 g/100 mL. Hematoxylin-eosin staining, 200X.

The histology of pancreatic islet cells presented normal morphology in non-diabetic and *C. papaya*-treated non-diabetic rats. Langerhans’ islets present in the pancreatic tissue featured circular shapes with healthy cell lining. The exocrine acini portions were well organized and with normal morphology (Figure 1a). The islet diameter in non-diabetic rats was 134.3±15.13 μm, whereas for treated non-diabetic animals for treated in non-diabetic rats were 163±19.36, 45.25±23.07 μm (0.75 and 1.5 g/100 mL, doses, respectively) (p< 0.05) (Figure 1b). In diabetic rats without treatment, the pancreatic tissue showed shrunken Langerhans’ islets (Figure 1c). The pancreatic tissue of *C. papaya-*treated diabetic rats exhibited an increased number of Langerhans’ islets in comparison to non-treated diabetic rats, suggesting a protective effect on the islets (p<0.05). Islet diameters in untreated diabetic rats were smaller in comparison to 0.75 and 1.5 g/100 mL *C. papaya*-treated diabetic rats (93.25±23.07, 89±13.07 vs. 61.88±12.86. μm; respectively) (Figure 1c, d and e) (p< 0.05).

**Figure 2:**
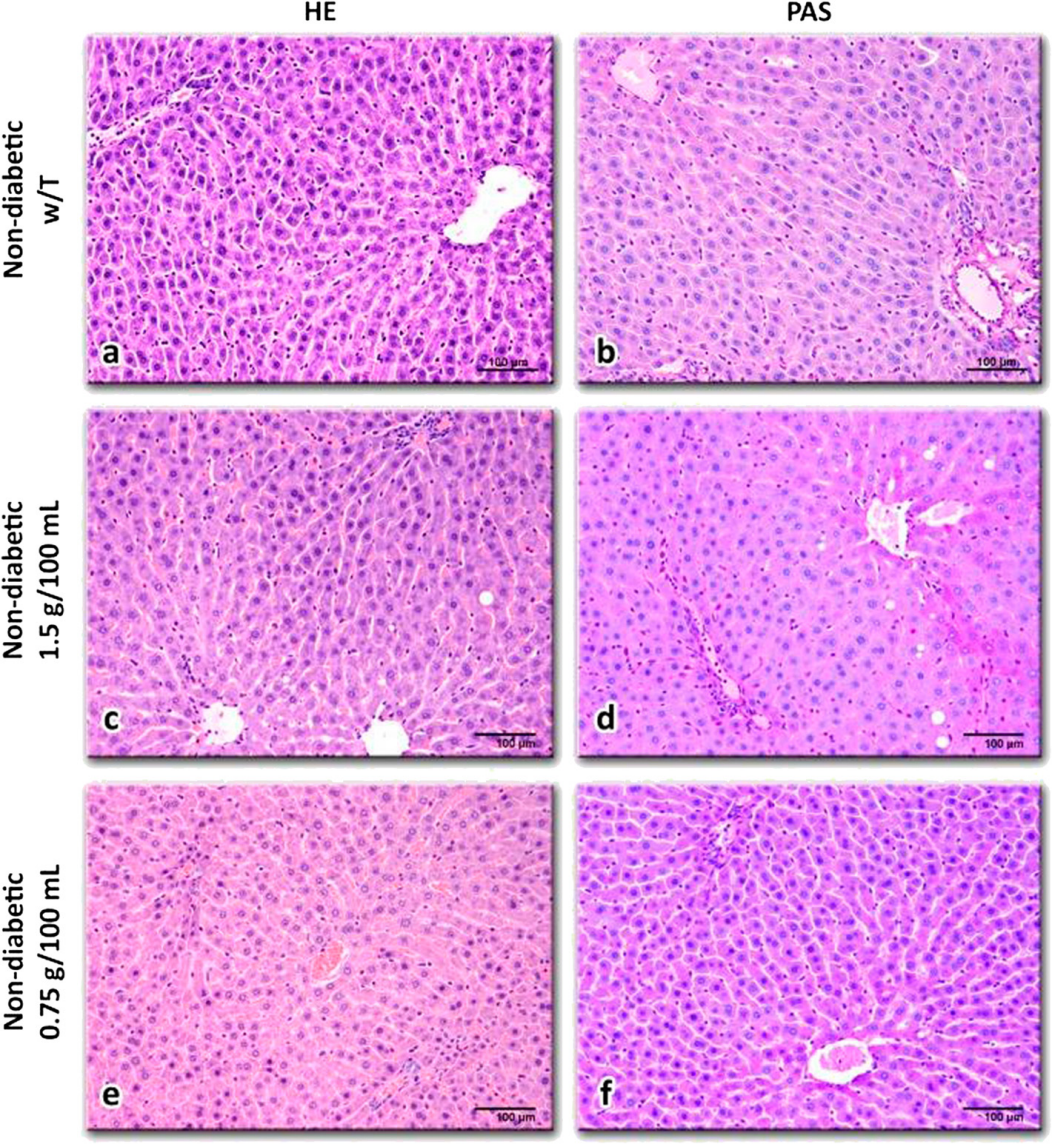
**Liver tissue slices from non-diabetic rats without treatment (a, b) and with *C. papaya *aqueous extract treatments: 1.5 g/100 mL (c, d) or 0.75 g/100 mL (e, f).** All liver sections showed normal structure; however, rat livers treated with 1.5 g/100 mL of *C. papaya* extract exhibited slight univesicular steatosis near the central veins. Glycogen content in all cases was insignificant. Hematoxylin-eosin (**a, c, e**) and PAS (**b, d, f**) staining, 200X.

**Figure 3:**
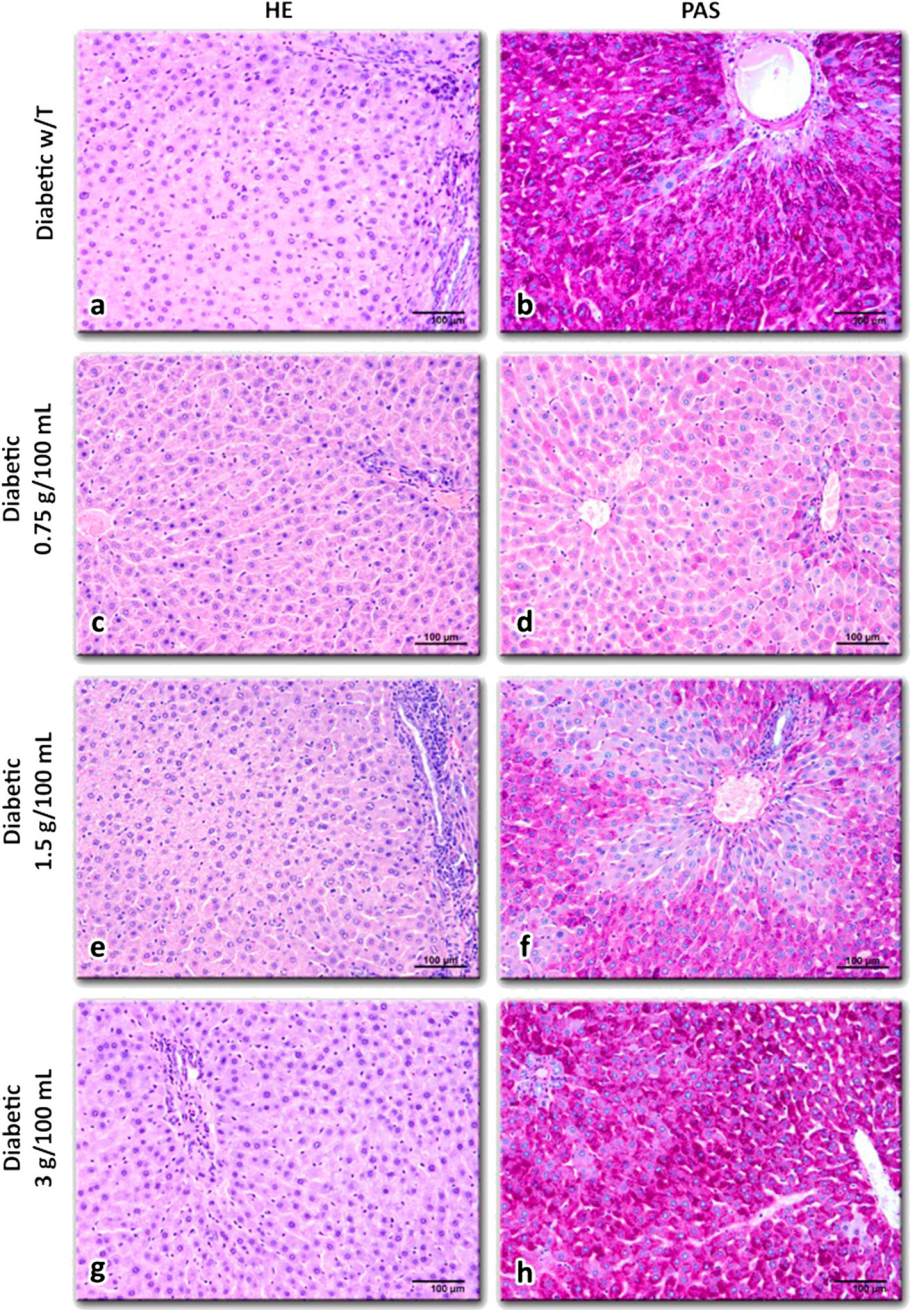
**Liver slices from diabetic rats without treatment (a, b) and with *C. papaya *aqueous extract treatments: 0.75 g/100 mL (c, d), 1.5 g/100 mL (e, f) or 3 g/100 mL (g, h).** It is noteworthy that in (**d**) and (**f**) the glycogen content in hepatocytes was reduced as compared with (**b**). **h**) Similar tissue aspect than in (**b**). Hematoxilin-eosin (**a, c, e, g**) and PAS (**b, d, f, h**) staining, 200X.

Hepatocytes in non-diabetic rats showed normal cell structure with well-preserved cytoplasm (Figure 2a), with small fat vesicles and glycogen granules in the cytoplasm (Figure 2b). The liver morphology of non-diabetic rats treated with *C. papaya* was similar to the control group (Figure 2c to f). On the other hand, the liver of diabetic rats showed differences: sinusoids were not clearly observed, abundant fat vesicles and glycogen granules especially around the central veins were present (Figure 3d and f). The liver morphology of diabetic rats treated with *C. papaya* (0.75 g/100 mL and 1.5 g/100 mL; Figure 3d and f, respectively) exhibited smooth changes compared to the untreated group, which entailed diminished disruption of hepatocytes (Figure 3c to f), smaller glycogen granules and fat vesicles content.

## Discussion

Our results show that the intraperitoneal administration of STZ to rats significantly increased glucose blood levels four days after injection, as well as decreased body weight. In addition, other diabetes-related signs were observed. These results agree with previous observations that have employed this model and that also report loss of body weight [13,14].

Several reports suggest that this model of type 1 diabetes induced by STZ is adequate to evaluate the properties of leaves or fruits from different plants [15-17]. In the present study, the aqueous extract of *C. papaya* maintained the body weight of diabetic rats during treatment. Weight loss is a main sign of diabetes but its mechanism is not clear. It could be due to many factors such as loss of appetite, increased muscle waste and loss of tissue proteins [15,18,19]. In addition, the administration of the aqueous extract of *C. papaya* decreased water consumption. Other natural products also produce a decrease in both water intake and urine volume excretion, indicating that these products can regulate water balance and which is known to depend on the kidney function [18,20]. In this study, however, kidney function biomarkers were not determined.

This study showed that the *C. papaya* leaf aqueous extract significantly diminished blood glucose levels (p<0.05) in diabetic rats. This hypoglycemic effect is similar to the one reported for other plants [17,18,21,22]. Such effect may be explained in part by either a decrease in the rate of intestinal glucose absorption [23-25] or an increase in peripheral glucose utilization [24,25]. In this line, some authors have ascertained increased catabolism of glucose due to GLUT4 translocation to the plasma membrane in muscle and brown adipose cells [19,20], with up-regulation of the uncoupling protein-1 in brown adipose tissue and hepatic gluconeogenesis [25,26], causing as a result hyperinsulinemia or enhancement of peripheral glucose utilization [19,27,28]. Moreover, a possible stimulatory mechanism on the few surviving β-cells has been considered, which could allow the release of more insulin [29-32]. Our results suggest that the aqueous *C. papaya* leaf extract may act by stimulating the few remaining β-cells with the subsequent release of more insulin, instead of pointing to the regeneration of β-cells of the islets as responsible for the insulin increase (Figure 1e).

Different reports have shown that the islets appear to be preferentially affected in diabetes by destruction of insulin-secreting β-cells [29,31,33]. In this respect, the damage to islets in diabetic rats treated with *C. papaya* extract was reduced. There are reports suggesting that in diabetic rats, the administration of plant extracts can be effective in cell regeneration and restoration of islet size, even producing cell hyperplasia [29,32,33]. β-cells have shown a remarkable potential for regeneration at the pre-clinical stage of diabetes which is a key question when addressing type 1 diabetes [29,30]. In addition, some authors using a pancreatic islet cell line have reported that plant extracts play a role in stimulating insulin secretion in β cells [20,22,32,33]; however, this does not seem to be the case for insulin-producing β-cells deteriorated by STZ.

We also found that insulin levels were diminished in diabetic rats compared to non-diabetic animals. This result is consistent with reports from other authors using STZ to induce type 1 diabetes in which they observed that STZ depletes insulin production by pancreatic β-cells [33,34]. Several authors suggest that the hypoglycemic or antidiabetic effect of some natural extracts can be attributed to their insulin-trophic effect that enables the reduction of blood glucose levels, liver glycogen content, and serum lipids through the control of serum insulin [20,30]. Interestingly, our results show that *C. papaya* extract increased insulin production, with no significant hypoglycemic effect on non-diabetic rats.

Our results demonstrated that triacylglycerol levels decreased in diabetic rats with the administration of *C. papaya* extract. The hyperlipidemia associated with diabetes may result from an accelerated hepatic triglyceride biosynthesis and the release of VLDL without an increase in its rate of clearance from the blood by lipoprotein lipase, which is dependent on the insulin/glucagon ratio [27,30]. Furthermore, the increase in lipid profile may be a consequence of increased lipids breakdown and mobilization of free fatty acids from peripheral deposits [30,32]. This biochemical evidence for the hypotriacylglycerolemic and hypoglycemic actions of *C. papaya* extract is supported by the improvement in the histological features of fat (Figure 3a) and glycogen (Figure 3b) content in hepatocytes of diabetic rats. The *C. papaya* extract decreased liver glycogen content in diabetic rats (Figure 3d,f). This diminution was not due to the action mediated by insulin because insulin levels were not increased in diabetic rats treated with *C. papaya.* It is known that in diabetes the activation of the gluconeogenic enzymes may also be due to the state of insulin deficiency given under that condition; insulin functions as a suppressor of gluconeogenic enzymes [33,35]. The inhibition of glucose intestinal absorption could have caused the significant reduction in liver glycogen. However, a direct effect of *C. papaya* extract on the activities of hepatic gluconeogenic enzymes cannot be ignored and need further investigation. Additionally, the consumption of herb extracts in diabetic rats reduced blood glucose levels and increased glucose tolerance not only by promoting insulin sensitivity, but also by reversed activity of hepatic enzymes in diabetic rats near to normal levels, through mechanisms that involve gluconeogenesis [35-37]. Several studies have revealed that increased total lipid, triglycerides and total cholesterol levels in the serum of diabetic rats are also found in liver and kidney [27,37]. Previous studies have reported that some phytocomponents, particularly saponins and steroids, elicit antihyperlipidemic action by inhibiting intestinal lipid absorption via resin-like action and inhibition of lipase activity [23,27,30]. On the other hand, the elevation of serum biomarker enzymes such as ALT, AST and ALP has been observed in diabetic rats indicating impaired liver function that may be due to hepatic damage induced by hyperglycemia [32-35]. In the present study, our results showed that *C. papaya* treatment produced a decrease in serum aminotranferases in diabetic rats. Liver damage in diabetic rats (Figure 3a and b) was confirmed, as well as improvement in hepatocyte morphology after the *C. papaya* treatment (Figure 3c to f). Moreover, reduced levels of total and direct bilirubin concentrations were observed in diabetic rats treated with *C. papaya.* Besides, biochemical evidence indicates that the increment in bilirubin concentration is generated by an enhanced liver function and muscle wasting. Furthermore, diabetes itself can induce injury to the bile ducts and cause muscle damage [22,23]. Similar results have been reported after the administration of *Croton cajura* extract in diabetic rats showing a decrease in other biochemical markers (transaminases, nitrogen and antioxidant enzymes in serum). This reduction led to a recovery in the metabolism of the diabetic rats and prevented the development of diabetic complications [27,32,37]. Our results suggest that the aqueous extract of *C. papaya* at low doses (0.75 and 1.5 g/100 mL) regulates bile transit and hepatic function in diabetic rats, but at high doses it can be hepatotoxic (3 g/100 mL). In this respect, there are reports of liver damage due to natural and drug treatments forcing discontinuation of treatment and the urgency of re-evaluating the pharmacokinetics and pharmacodynamics of these compounds [35-37].

In our study, there is evidence of a reduction in NO metabolites in diabetic rats [38,39]. In addition, the administration of the aqueous extract of *C. papaya* to diabetic rats increased NO levels. As it is well known, diabetes is characterized by hyperglycemia and hyperlipidemia, two biochemical features associated with inhibition of endothelial nitric oxide synthase (eNOS), leading to diminished NO production, increased formation of reactive oxygen species (ROS), impaired endothelium-dependent relaxation, increased formation of free radicals and lower efficacy of antioxidant systems, which lead to an imbalance between free-radical formation and the protection against them [32,38-40]. However, the presence of antioxidant molecules regulating NO production generates a diminution in oxidative stress [38-40]. Several studies have reported that medicinal plant extracts have flavonoids, saponines and polyphenols that increase the activity of antioxidative systems [40,41]. This antioxidant effect of plant extracts decreases the oxidative stress generated by diabetes, resulting in a reduced or delayed progression of the endothelial degeneration, nephropathy and neuropathy [38-41]. In this sense, the antidiabetic effect of *C. papaya* extract can be due to its content of chemical constituents responsible for antioxidant actions.

Data are preliminary on the hypoglycemic effect of *Carica papaya* leaves in streptozotocin-induced diabetic rats. This study have some limitations: a sample size with six animals in every group, a short period of study, the diabetes model correspond more to a type 1 diabetes than to type 2 diabetes, moreover the active metabolite in the *C. papaya* leaves was not identified. Further studies administering the extract for longer periods of time are necessary.

Taken altogether, these results show that the administration of the extract of *C. papaya* leaf induced a significant reduction in glucose and triacylglycerol plasma concentrations (0.75 and 1.5 g/100 mL). In addition, this extract exhibited an antioxidant action and was not hepatotoxic at low doses (0.75 and 1.5 g/100 mL). The suggested mechanism for *C. papaya* could be similar to that reported for some sterols which decrease the activity of lipid- and carbohydrate-hydrolyzing enzymes in the small intestine, thus reducing the conversion of disaccharides and triglycerides into absorbable monosaccharides and free fatty acids [23,31]. Currently, there are few reports on the effect of papaya leaves in experimental diabetes. In recent years, the use of therapeutic phytoproducts has been consistent; however, multicenter, large-scale clinical trials are needed to evaluate their safety and efficacy, as well as their interaction with conventional drugs when administered simultaneously.

## Conclusions

This preliminary study confirms the hypoglycemic effect of *C. papaya* leaves together with other beneficial effects in diabetic rats. These results suggest that the aqueous extract of *C. papaya* may improve the metabolic disruption produced by diabetes. However, further research is needed to gain a better understanding of its potential therapeutic action, the implicated phytochemical constituents and the exact mechanism of action.

## Abbreviations

Chol: Cholesterol; TAG: Triacylglyceride; HDL-Ch: High-density lipoprotein-cholesterol; NOx: Nitric oxide concentration; NO_3_+NO_2_: Nitrate+nitrite; AST: Aspartate aminotransferase; ALT: Alanine aminotransferase; ALP: Alkaline phosphatase; TB: Total bilirubin; DB: Direct bilirubin; LW: Liver weight.

## Competing interests

The authors declare that they have no competing interests.

## Authors’ contributions

JRIE, DZJC and BODY conceived the study, participated in its design, and helped to draft the manuscript. CRAE, TZC and BCJL helped to perform the statistical analysis and to draft the manuscript. RHA and RFT coordinated and supervised the integration of data. MOPH and AMH helped with the integration of data and analysis. All authors read and approved the final manuscript.

## Pre-publication history

The pre-publication history for this paper can be accessed here:

http://www.biomedcentral.com/1472-6882/12/236/prepub
